# Similar genomic patterns of clinical infective endocarditis and oral isolates of *Streptococcus sanguinis* and *Streptococcus gordonii*

**DOI:** 10.1038/s41598-020-59549-4

**Published:** 2020-02-17

**Authors:** Katrine Højholt Iversen, Louise Hesselbjerg Rasmussen, Kosai Al-Nakeeb, Jose Juan Almagro Armenteros, Christian Salgård Jensen, Rimtas Dargis, Oksana Lukjancenko, Ulrik Stenz Justesen, Claus Moser, Flemming S. Rosenvinge, Xiaohui Chen Nielsen, Jens Jørgen Christensen, Simon Rasmussen

**Affiliations:** 1 Department of Health Technology, Section for Bioinformatics, Technical University of Denmark, Kemitorvet, Building 204, 2800 Kgs. Lyngby, Denmark; 2 The Regional Department of Clinical Microbiology, Region Zealand, Ingemannsvej 46, 4200 Slagelse, Denmark; 3 Clinical Microbiomics, Ole Maaløes vej 3, 2200 Copenhagen N, Denmark; 4 Department of Clinical Microbiology, Rigshospitalet, Henrik Harpestrengsvej 4A, 2100 Copenhagen Ø, Denmark; 5 Department of Clinical Microbiology, Odense University Hospital, J.B. Winsløws Vej 21, 2, 5000 Odense C, Denmark; 6 Institute of Clinical Medicine, University of Copenhagen, Blegdamsvej 3B, 2200 Copenhagen N, Denmark; 7 Novo Nordisk Foundation Center for Protein Research, Faculty of Health and Medical Sciences, University of Copenhagen, Blegdamsvej 3B, 2200, Copenhagen N, Denmark

**Keywords:** Comparative genomics, Next-generation sequencing, Bacterial infection

## Abstract

*Streptococcus gordonii* and *Streptococcus sanguinis* belong to the Mitis group streptococci, which mostly are commensals in the human oral cavity. Though they are oral commensals, they can escape their niche and cause infective endocarditis, a severe infection with high mortality. Several virulence factors important for the development of infective endocarditis have been described in these two species. However, the background for how the commensal bacteria, in some cases, become pathogenic is still not known. To gain a greater understanding of the mechanisms of the pathogenic potential, we performed a comparative analysis of 38 blood culture strains, *S*. *sanguinis* (*n* = 20) and *S*. *gordonii* (*n* = 18) from patients with verified infective endocarditis, along with 21 publicly available oral isolates from healthy individuals, *S*. *sanguinis* (*n* = 12) and *S*. *gordonii (n* = 9). Using whole genome sequencing data of the 59 streptococci genomes, functional profiles were constructed, using protein domain predictions based on the translated genes. These functional profiles were used for clustering, phylogenetics and machine learning. A clear separation could be made between the two species. No clear differences between oral isolates and clinical infective endocarditis isolates were found in any of the 675 translated core-genes. Additionally, random forest-based machine learning and clustering of the pan-genome data as well as amino acid variations in the core-genome could not separate the clinical and oral isolates. A total of 151 different virulence genes was identified in the 59 genomes. Among these homologs of genes important for adhesion and evasion of the immune system were found in all of the strains. Based on the functional profiles and virulence gene content of the genomes, we believe that all analysed strains had the ability to become pathogenic.

## Introduction

The oral cavity is covered by a mixed-species biofilm. The composition of the biofilm, as well as the abundance of specific species has a great impact on the oral health^1^. Among the pioneer colonizers of the oral cavity, we find *Streptococcus sanguinis* and *Streptococcus gordonii*^2,3^. Both species are non-hemolytic streptococci and commensal members of the Mitis group. They are found to prevent dental caries by inhibiting the bacterial growth of the plaque forming Mutans streptococci^1,4^.

The oral cavity is an extreme environment; the bacteria have to cope with variations in temperature and pH, oxidative stress and strong hydrodynamic as well as mechanical forces caused by food consumption, chewing, talking and movement of the tongue^1^. To form a biofilm in such an environment, the bacteria need to adhere to the surface of the host and to each other. Furthermore, the Mitis group streptococci species have developed the ability to interact and modulate the cells from the human immune system. This enables the bacteria to escape detection and may hinder an immune response provided by the immunoglobulins^4–6^. Natural genetic transformation, by uptake as well as release of DNA, has been described in several species belonging to the Mitis Group streptococci^1,7^. This genetic competence is an important mechanism for acquiring genes involved in biofilm formation, adherence and resistance to host immune systems^7–9^.

Even though the natural habitat of *S*. *gordonii* and *S*. *sanguinis* is the oral cavity, the bacteria can escape their niche and cause severe infections as infective endocarditis (IE)^2,10^. Infective endocarditis is a relatively rare infectious disease with an incidence of around 1.7–6.2 per 100,000 patients each year in the USA and Europe^11^. Despite its rarity, IE is a disease with a high mortality rate of approximately 40%^11^. The treatment often requires long antibacterial therapy, surgery and as a result, long-term hospitalisation^12^. The rate of IE cases caused by oral non-hemolytic streptococci varies globally from 17–45%^12,13^.

In recent decades, researchers have tried to elucidate the mechanisms that turn *S*. *sanguinis* and *S*. *gordonii* into pathogens. Especially proteins related to adhesion and the contribution of the evasion of the immune system have been given special attention. Genomic comparison of strains isolated from patients with IE and oral strains may shed light on what triggers the bacterium to become pathogenic.

When comparing multiple homolog protein sequences some regions in the sequence are more conserved than others^14^. These conserved regions are often referred to as protein domains, which are fundamental units of the structure and evolution of the proteins^15^. A protein can contain one or more domains, and the domain architecture has great importance for the tertiary structure and therefore also the function of the protein^16^.

Using whole genome sequencing data, we are able to predict functional domains in the translated genes. By comparing these functional domain architectures of 27 *S*. *gordonii* and 32 *S*. *sanguinis* genomes, constructing phylogenetics based on amino acid variations in the translated core genome and applying machine learning, we were able to make a clear separation of the two species. The analysis revealed species-specific genomic patterns of *S*. *gordonii* and *S*. *sanguinis*. In addition, we attempted to identify genomic patterns that could distinguish the 38 clinical IE isolates from the 21 oral isolates from healthy individuals. However, we were not able to identify any clear genomic signatures within the clinical IE genomes that could be used to distinguish them from the oral isolates. We identified several virulence genes that could contribute to the pathogenesis of the bacteria in both the IE isolates and oral isolates. Our results therefore suggest that all analysed strains had the ability to become pathogenic.

## Results

### *De novo* assembly of *S. gordonii* and *S. sanguinis*

We assembled the sequence data obtained from Illumina sequencing of 27 *S*. *gordonii* and 32 *S*. *sanguinis* strains into relatively few scaffolds (Table 1) (6–30 scaffolds). In comparison the assemblies we downloaded from NCBI^17^ ranged from 1–53 (9 *S*. *gordonii*) and from 1–163 (12 *S*. *sanguinis*) scaffolds, which was in compliance with our own assemblies. The estimated sizes of the genomes ranged from 2.1 Mb–2.3 Mb (*S*. *gordonii*) and from 2.3–2.4 Mb (*S*. *sanguinis*) for both the IE and oral genomes. Between 2,018–2,281 coding sequences (CDSs) were predicted in the *S*. *gordonii* strains and 2,183–2,386 CDSs were predicted in the *S*. *sanguinis* strains (Table 1), which are within the expected values obtained from already published strains. The strain ID, number of scaffolds, N50 and GC% in the assemblies of the 59 *S*. *gordonii* and *S*. *sanguinis* genomes are presented in the supplemental material (Supplementary Table S1).

**Table 1:** Species, isolation source, number of isolates, number of scaffolds, genome size and coding sequences.

Species	Isolation source	#isolates	#scaffolds	Genome size	Coding sequences
*S*. *sanguinis*	Clinical IE isolate	20	6–30	2.3–2.4 Mb	2,183–2,386
	Oral isolate	12	1–163		2,199–2,321
*S*. *gordonii*	Clinical IE isolate	18	10–29	2.1–2.3 Mb	2,068–2,171
	Oral isolate	9	1–53		2,018–2,167

### 134 protein families were found to be specific for the species

**Figure 1:**
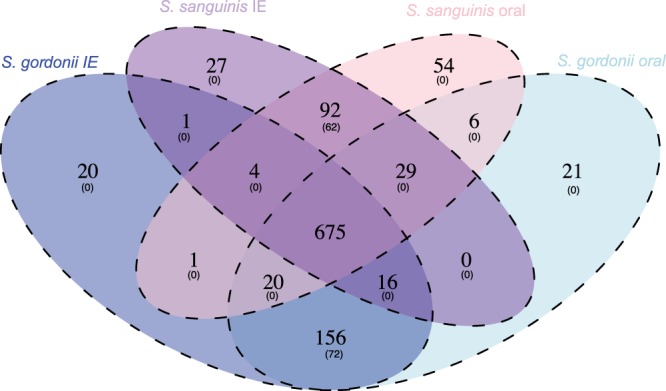
Venn-diagram showing the number of unique protein families as well as the number of proteins exclusively (number in parentheses) shared between the four different groups: *S*. *gordonii* IE isolates (dark blue), *S*. *gordonii* oral isolates (light blue), *S*. *sanguinis* IE isolates (dark purple), *S*. *sanguinis* oral isolates (light purple), and their overlapping groups. The centre of the diagram, where all four groups overlap, is considered as the common core-genome.

In each of the genomes, we identified protein domains from predicted and translated CDS. The translated CDS with identical protein-domain content and architecture were assigned to the same protein family. Translated CDS, for which no protein domains could be identified, were clustered based on sequence homology and each cluster was considered as a protein family. A total of 4,476 protein families were identified in the pan-genome, whereas 675 protein families were identified as common core-genes, as they were present in the 59 streptococci genomes. All possible combinations of shared core genes were calculated for the four groups: clinical IE and oral *S*. *gordonii* genomes, and clinical and oral *S*. *sanguinis* genomes. We only identified one core-gene shared between the clinical IE *S*. *sanguinis* and *S*. *gordonii* genomes. This gene was not exclusive to the clinical strains; it was also found in some of the oral genomes. The presence of the core-gene in the clinical IE strains and in some of the oral strains indicates the potential of this to be an important virulence gene. The core-gene contained the two functional domains, PF01071 and PF04262, with the functions phosphoribosylamine-glycine ligase and glutamate-cysteine ligase activity, respectively. These two enzymes carry out the second step in purine biosynthesis and the first step of the glutathione biosynthesis pathway^18^. Similarly, we identified six core-genes specific to the oral strains. Even though these genes were present in all oral strains, they were also found in some of the clinical IE isolates. More core-genes were found within the two species independent of clinical status (Fig. 1). Of the 92 unique *S*. *sanguinis* core-genes, 62 were not found in any of the *S*. *gordonii* isolates. Additionally, 72 of the 156 unique core-genes of *S*. *gordonii* were absent in all the *S*. *sanguinis* isolates. This means that it is possible to separate the two species based on presence or absence of specific genes. None of the genes seemed to be specific for the IE isolates or the oral isolates. The presence or absence of single genes could therefore not be used to distinguish between pathogenetic and potential pathogenic isolates.

### Clinical IE or oral isolates are phylogenetic alike

**Figure 2:**
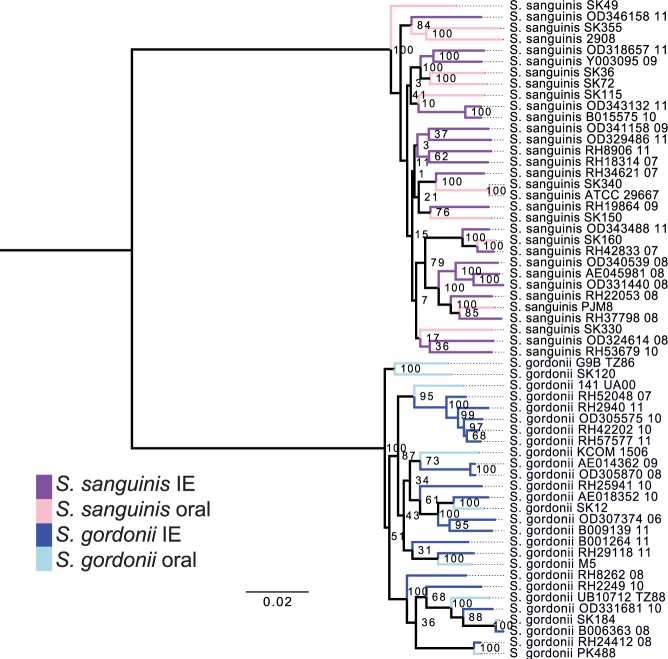
Phylogenetic tree of the 59 streptococci genomes, constructed on the basis of the protein sequences of 675 core-genes. The tree is obtained by PhyML using the JTT amino acid substitution model and bootstraping of 100 runs (the bootstrap values are shown in the tree). The scale bar indicates the evolutionary distance between the sequences determined by 0.02 substitutions per amino acid position. The colours in the figure indicates the four different groups of isolates: *S*. *gordonii* IE isolates (dark blue), *S*. *gordonii* oral isolates (light blue), *S*. *sanguinis* IE isolates (dark purple), and the *S*. *sanguinis* oral isolates (light purple).

**Figure 3:**
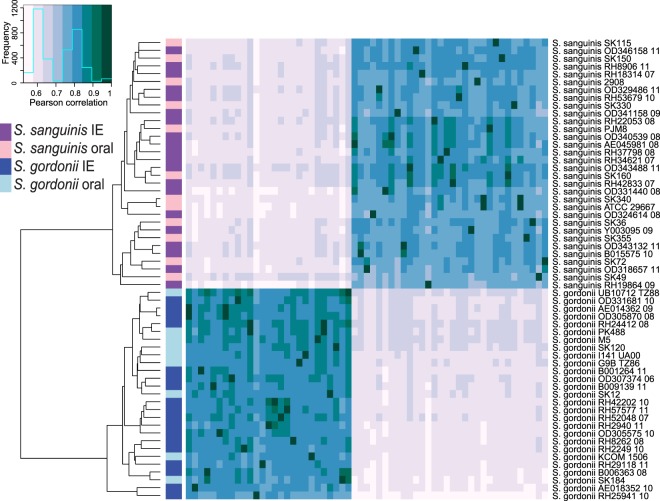
Hierarchical clustering of Pearson correlation coefficients determined from the absence or presence of the individual protein families in each of the analysed genomes. The heatmap colours indicate the Pearson correlation coefficient among the strains: The colormap in the left corner illustrates the correlation values by colour, as well as the frequency of each correlation value. The colour bar shows the individual species of each particular strain: *S*. *gordonii* IE isolates (dark blue), *S*. *gordonii* oral isolates (light blue), *S*. *sanguinis* IE isolates (dark purple), and the *S*. *sanguinis* oral isolates (light purple).

We reconstructed the phylogeny of the isolates using amino acid variations in the 675 common core-genes (Fig. 2). The phylogenetic tree was separated in two distinct clades consisting of the two species, yet no clades containing only IE or oral isolates were found. In addition, we clustered the strains using hierarchical clustering of Pearson correlation coefficients based on the absence or presence of protein families present in each strain (4,476 unique protein families in total). Similar to the core-genome tree there was a clear separation of the two species (Fig. 3). Thus, a clear separation of the two species could be made, no clear clustering patterns could be found between the IE and oral isolates. This implies that the variance on sequence level in the core-genome and gene content in the pan-genome was not sufficient to distinguish the clinical IE genomes from the oral genomes. To further investigate whether specific genes would separate between the clinical IE and oral isolates we reconstructed the phylogeny based on each of the 675 core genes. Here 464 of the gene trees could separate the isolates on species level, however, none of the individual phylogenetic trees were able to separate the clinical IE from the oral genomes. Therefore, per gene sequence variation were not able to determine the clinical origin of the isolates.

### Machine learning predicts a similar pattern in clinical IE and oral genomes

Due to the lack of separation between the clinical IE and oral genomes, we applied machine learning in the form of a Random Forest (RF) to identify combinations of genes that could distinguish these two groups. We constructed two datasets: one using the absence and presence of the protein families as described above, and another where we summed up the number of the individual protein domains in each genome (4,490 domains and clusters in total). To decrease the strong species specific signal we removed redundancy in the dataset. This reduced the dataset to 1,868 features when using counts of the functional domains and 1,540 features when using the presence or absence of protein families.

**Figure 4:**
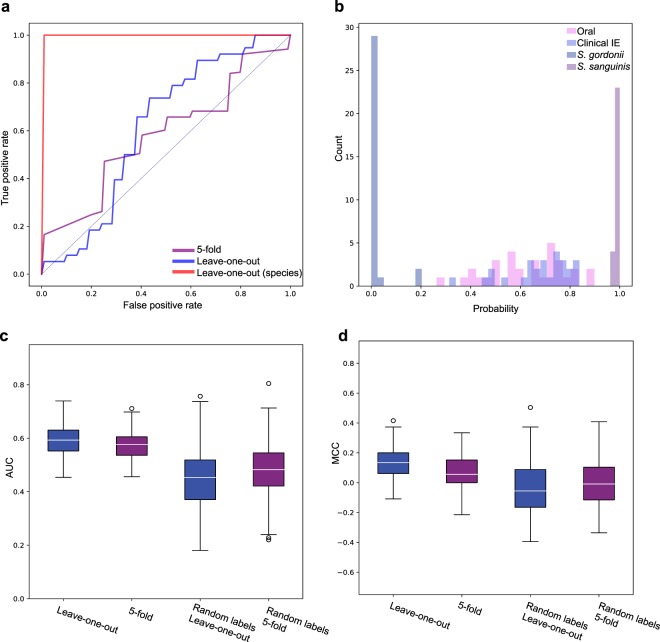
Machine learning using Random Forest modelling based on the none redundant count of individual protein domains in each genome. (**a**) ROC curves for the species model using LOU CV (red), the clinical vs. oral model using LOU CV (blue) and 5-fold CV (purple). (**b**) Histogram of the prediction probabilities for the LOU CV  of the species and clinical vs. oral model. (**c**) Boxplots of the AUC determined from 100 runs using LOU (blue) and 5-fold CV (purple) on the clinical vs. oral model. The boxplots also show the values when random labelling is applied. (**d**) Boxplots of MCC determined from 100 runs using LOU (blue) and 5-fold CV (purple) on the clinical vs. oral model. The boxplots also show the values when random labelling is applied. Boxplots shows the distribution of the data by illustrating the minimum and maximum values, as well as the first and third quartile (the box) with the median highlighted with white. Outliers are illustrated as circles outside of the plot.

Using a stratified leave-one-out (LOU) cross-validation (CV) approach, we trained the Random Forest model to separate the *S*. *gordonii* from the *S*. *sanguinis*. As expected, there was a very strong prediction signal with a mean Area Under Curve (AUC) of 1.0 and mean Mathews Correlation Coefficient (MCC) of 1.0. The Receiver Operating Characteristic (ROC) curve was furthermore found to be close to perfect (Fig. 4a). The AUC was significantly better compared to random labelling of the strains (t-test, p-value = 0.002). The model clearly separated the *S*. *gordonii* from the *S*. *sanguinis* strains. None of the strains were misclassified as illustrated in the histogram in Fig. 4b (the model predicts the strains to be *S*. *gordonii* when probability <0.5, and *S*. *sanguinis* when probability >0.5).

We used the same approach as described above in an attempt to distinguish the clinical IE from the oral isolates. We achieved a ROC curve slightly better than the random performance level (Fig. 4a) and a mean AUC of 0.58 (Fig. 4c), however mean MCC was 0.09, indicating that the model was random guessing (Fig. 4d). The probabilities of the predictions were centred around 0.4–0.8 (Fig. 4b) indicating that the model cannot clearly distinguish the two groups and tend to predict the isolates as clinical IE isolates. This indicates that there is a high false-positive-rate likely caused by a bias in the number of pathogenic strains in our dataset (38:21). We furthermore applied one-hot encoding which did not improve the predictions (mean AUC of 0.51, and mean MCC of −0.01) (Supplementary Fig. S1).

Finally, to investigate if amino acid variation could separate the clinical IE isolates from the oral isolates, we extracted all variable positions based on the core-genome (32,675 residues). We, however, found no residues that were identical or near identical (up to 2 differences) between all strains of either the clinical IE or oral isolates, whereas 6,140 residues (18.8%) were specific when comparing the two species. When applying RF on the 21,165 unique amino-acid variations, we found the performance to decrease significantly compared with the two previous datasets. Here we got a mean AUC of 0.47 and a mean MCC of −0.05 (compared to counts of the functional domains: p-value < 2.2e-16, compared to one-hot-encoding: p-value = 3.48e-07) (Supplementary Fig. S1).

### The virulence genes found in all 59 strains

**Figure 5:**
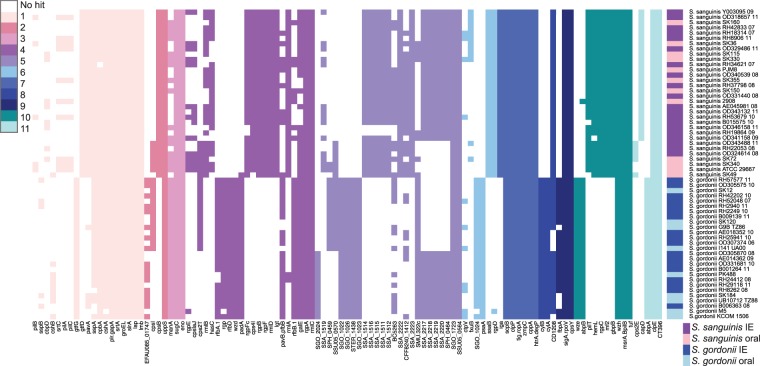
Identification of virulence factors from the Virulence Factor Data Base (VFDB) using both the core and the full dataset of the database. The heatmap colours indicate 11 different groups of virulence genes (the white spaces mean that no hit was found). The categories of the virulence genes can be found in the colour bar to the right of the heatmap and are: (1) Adherence, (2) Antiphagocytis, (3) Carbonhydrate transport and metabolism, (4) Cell wall/membrane/envelope biogenesis, (5) Immune evasion, (6) Inorganic ion transport and metabolism, (7) Protease, (8) Toxin, (9) Transcription, (10) Other, (11) Uncategorized. The second colour bar in the heatmap show the individual species of each particular strain: *S*. *gordonii* IE isolates (dark blue), *S*. *gordonii* oral isolates (light blue), *S*. *sanguinis* IE isolates (dark purple), and the *S*. *sanguinis* oral isolates (light purple). Genes with less than 6 hits were not illustrated in the figure but can be found in Supplementary Table S2.

Even though we could not distinguish the clinical and oral isolates based on whole genome analyses, we investigated the presence of known virulence genes. Therefore we aligned all genes from the 59 genomes using BLAST^19^ to the Virulence Factor Data Base (VFDB)^20–22^ core database as well as the full database. We found 3,948 hits across 151 different virulence genes. Some of the virulence genes were homologs but gave hits in different species. These homolog virulence genes were merged together as one hit, yielding 3,808 hits across 118 virulence genes. Using VFDB’s own classification as well as Clusters of Orthologous Groups (COGs)^23^ we divided the virulence genes in 11 different classes (Fig. 5 and Supplementary Table S2). We identified 26 different virulence genes that were present in all 59 genomes – thus the core genome. Among the 26 core-virulence genes, six were involved in adherence (*groEL*, *lap*, *lmb*, *slrA*, *strA*, *plr/gapA*). Since bacterial adhesion and colonization of the heart valves is a prerequisite for development of IE^24^, these six core genes could contribute to the development of bacterial IE. Enolase encoded by *eno*, which was also found in the core genome, contributes to complement evasion. The enzyme binds *S*. *pneumoniae*, *S*. *sanguinis* and *S*. *gordonii* strains to human plasminogen and thereby promote development of IE^25,26^. This gene could therefore have an influence on the pathogenicity of the strains and the development of IE. We furthermore investigated whether any virulence genes were exclusive to either of the two species. In total 14 virulence genes were unique for *S*. *sanguinis* and 12 virulence genes were unique for *S*. *gordonii*, indicating different virulence mechanisms for the species (Fig. 5). None of the identified virulence genes were exclusive for the clinical isolates. This was additionally reflected by clustering the absence and presence of virulence genes where the isolates clustered according to species (Fig. 5). We observed no clustering of the oral or clinical IE isolates.

### Genes coding for immune evasion were part of the core genome

Immunoglobulin A1 (IgA1) protease encoded by *iga* (also known as *zmpA*) circumvents the immune system in the oral cavity by cleavage of the human immunoglobulin A1^27,28^ and IgA1 activity has previously been found to be present in *S*. *sanguinis* strains and absent in *S*. *gordonii*^29,30^. In this study, *iga* was only identified in the *S*. *sanguinis* core-genome (Fig. 5). We investigated the polysaccharide capsule (CPS) as it is indispensable for the virulence of *S*. *pneumoniae* by forming an inert shield preventing phagocytosis^31,32^. Additionally, the *cpsA-cpsD* operon has been found essential for encapsulation and regulation of CPS production^33^. The presence of a complete *cps* loci have been observed in several species in the Mitis group streptococci, including *S*. *sanguinis* and *S*. *gordonii*^34–36^. We have previously identified genes homologous to *S*. *pneumoniae* TIGR4 *cps4* in the core and pan-genome of *S*. *mitis* and *S*. *oralis*^37^. Using a database containing the *S*. *pneumoniae* TIGR4 *cps4* genes, we identified *cps4A-D* in all 59 genomes (Supplementary Table S3). The presence of the four *cps4* homologs in the core-genome, therefore, indicates that both the clinical IE and oral isolates could be encapsulated.

## Discussion

Genomic comparison and identification of virulence factors with importance for IE in *S*. *gordonii* and *S*. *sanguinis* have mostly been conducted using single or a few strains^38–41^. A previous study, by Zheng *et al*., used 19 genomes of *S*. *sanguinis* and *S*. *gordonii* for genomic comparison^41^. In this study, we increased the number to 59 genomes. Comparison of a larger number of genomes provides a deeper insight into the functional genomic patterns and significance of different known virulence factors that could contribute to development of IE.

The genome sizes, number of genes, and GC% are consistent with previous studies^41–44^. A total of 4,476 protein families were identified in the pan-genome of the 59 genomes. This was 75 additional pan-genomic families compared to the study of Zheng *et al*.^41^, which is surprisingly few, since this study included three times the number of genomes. Previous studies have shown that *Streptococcus* has an open pan-genome and frequent recombination that leads to gene gain and loss within Streptococcus^8,41,45^. Therefore, the size of the pan-genome is expected to increase as the number of genomes increases. However, differences in the methods to divide the proteins into families are used; Zheng *et al*.^41^ used RAST for gene calling and FIGfams to assign the functional proteins^41,46,47^. The core-genome size found in this study is more consistent with a previous study of 80 Mitis group streptococci clinical isolates from six different species, where 591 core-genes were identified^48^.

By investigating the pan-genome we hoped to identify genes that were only present in the oral isolates and other genes only present in clinical IE isolates. However, no genes were found to be specific to the isolation source. Few core-genes were found in IE isolates as well as all oral isolates, however, none of these core-genes were found to be unique for any of these two groups. This means that none of the pan-genes seemed to be specific for the IE isolates or the oral isolates. Nevertheless, several species-specific genes were identified. A total of 62 core-genes were found uniquely in *S*. *sanguinis*, while 72 core-genes were unique in *S*. *gordonii*. This result is consistent with the clades seen in the phylogenetic core-tree and in the clustering patterns of the heatmap; a clear separation of the species was observed, but no clear clustering patterns were found between the IE and oral isolates. The Random Forest modelling finds a weak signal for separating the clinical IE genomes from the oral genomes. However, the model tends to predict most of the genomes as clinical IE genomes, resulting in a fairly low accuracy.

Several studies have suggested that recombination, e.g. gain and loss of genes, has a greater importance for the pathogenic potential for a bacterium, than mutations in specific genes^49–52^. This study included both amino acid variation in the translated core genome as well as genetic information in the translated pan and core genome. Using hierarchical clustering and machine learning, no clear patterns were found to distinguish the oral from the clinical isolates. Nonetheless, the approaches were able to clearly separate the species.

We identified 118 known virulence genes in our 59 *Streptococcus* genomes. The presence of the specific virulence genes did separate the species in two distinct clusters. Homologs of genes contributing to immune evasion (*cps4A-cps4D* and *iga*) and adhesion (*groEL*, *lap*, *lmb*, *slrA*, *strA*, *plr/gapA*) were identified in the core-genome of the 59 *S*. *sanguinis* and *S*. *gordonii* genomes. The presence of these genes in the core-genomes indicates their importance for *S*. *sanguinis* and *S*. *gordonii* and could be important for their pathogenesis.

In summary, our results imply that it is probably not the function of a single gene that makes the bacterium pathogenic. The amino acid variations, functional profiles, as well as the virulence gene content of the isolates are prone to be more specific to the species, than to the isolation source. We therefore believe that the infection event is most likely very complex, where the health status and hygiene habits of the host is of great importance; diseases as diabetes, cancer and congenital heart disease has shown to be major risk factors for infective endocarditis^11^. Moreover, the interaction between the host immune system and the bacteria might be of importance for the development of the infection. Our study shows species specific virulence patterns, which implies that *S*. *gordonii* and *S*. *sanguinis* could have different virulence mechanisms. The absence of specific gene as well as virulence patterns within the clinical IE group suggest that all the analysed *Streptococcus* genomes carry similar pathogenic potential.

## Methods

### Bacterial strains

Thirty-eight blood culture strains, *S*. *sanguinis* (*n* = 20) and *S*. *gordonii* (*n* = 18) from patients with verified IE were collected retrospectively (2006–2013) from the Capital Region of Denmark (RH strains), Region Zealand (AE, Y and B strains) and Region of Southern Denmark (OD strains). The bacterial DNA was extracted as described in Rasmussen *et al*.^48^ and paired-end sequenced using Illumina Hiseq. 2000 with 100X coverage (BGI-Tech Solutions, Hong Kong, China). The draft genomes were assembled with SPAdes version 3.1.1^53^, using the following k-mers: 25,35,45,55,65,75,85,95, and the ‘careful’ mode to reduce assembly errors. Statistics regarding the assembly performance were achieved using scripts from the Assemblathon^54^. Species identification was based on Multi Locus Sequence Analysis (MLSA), Single Nucleotide Polymorphisms (SNPs) and core-genome phylogeny as described in Rasmussen *et al*.^48^. Additionally, we included 21 genomes isolated from healthy individuals from the oral cavity of *S*. *sanguinis* (n = 12) and *S*. *gordonii* (n = 9). These genomes were downloaded September 4^th^ 2017 from NCBI^17,55^ (the accession numbers, assembly ID and assembly statics of the oral isolates as well as the ENA accession number and the assembly statics of the clinical IE isolates can be found in Supplementary Table S1).

### Prediction of functional profiles

The pipeline PAN-genome analysis based on FUNctional PROfiles (PanFunPro)^56^ was used for gene prediction and for prediction of functional domains in the *de novo* assembled genomes. First genes were predicted and translated into protein sequences using prodigal v2.6.2^57^, where only closed genes were considered. The translated gene sequences in each streptococcal genome was searched against the databases; PfamA^18^, TIGRFAM^37^ and SUPERFAMILY^58^ using InterProScan software v. 5.25–64.0^59^ for prediction of functional domains. Genes with no identified functional domains were clustered using CDhit^60,61^ based on sequence similarity; a search window of five amino acids was used and proteins with a sequence similarity of 60% were assigned to the same protein family. Genes with identical functional domain architecture and genes belonging to the same gene cluster were considered to belong to the same protein family.

### Core-genome analysis

The core-genes for each strain were identified by selecting translated gene sequences from the protein families identified above which were present in all 59 genomes. To ensure homology of these genes, CD-HIT^60,61^ was used for clustering of the translated gene sequences, with a threshold for homology of 60% identity and 60% coverage of the query. Translated gene sequences complying with these criteria were aligned using MUSCLE v. 3.8.42^62^ and alignments containing less than 35% conserved sites or an average identity less than 80% were discarded. All verified core-genes were then considered as a part of the core-genome.

### Phylogeny and strain clustering

The pan-genome was retrieved using PanFunPro^56^ by considering all unique protein profiles and gene clusters in the 59 genomes as a part of the pan-genome. A matrix was generated based on the absence or presence of the individual protein families in each of the analysed genomes. The matrix was used to generate a heatmap with hierarchical clustering of Pearson correlation coefficients to determine clustering patterns between the genomes (R v. 3.2.3^63^ with the gplots^64^ library). All alignments were concatenated using an in-house python script (available at https://github.com/RasmussenLab/StreptococcusMitis), and a phylogenetic tree was reconstructed using PhyMl v. 3.1^65^, using the JTT amino acid substitution model and bootstrapping of 100 runs as described in Rasmussen *et al*.^48^. Furthermore, a phylogenetic tree was generated using the same settings, on each of the core-gene alignments, individually.

### Machine learning for virulence prediction

Python v. 3.6.1 was used for machine learning modelling with the libraries: matplotlib v. 2.0.2^66^ for plotting, Scikit-learn v. 9.0.1^67^ for Random Forest, K-fold splitting and correlation calculations. Pandas v. 0.20.1^68^ and numpy v. 1.13.1^69^ for handling the data and removing redundancy. Random Forest modelling was applied to two different matrices extracted for the pan-genome of the 59 genomes; (1) an absence-presence matrix of all 4,476 protein families, which were reduced to 1,540 features by removing redundancy, (2) a count matrix of each of the unique functional domains and identified clusters individually, yielding in 4,490 different functional domains and clusters. This matrix was reduced to 1,868 features when removing redundancy. The settings used in the 5-fold cross-validation (CV) model: number of splits = 5, random state = 100, shuffle = True, number of estimators = 10, max features = None, max depth = 3. Leave-one-out (LOU) CV: number of splits = 59, random state = 100, shuffle = True, max features = None, estimators = 10, max depth = 3. For consistency, the random seed of 18 was used in the analysis in Python using the Random library. To reduce redundancy and the strong species specific signal, all duplicates in the dataset was removed.

Labelling was applied to the model, where y = 1 for clinical IE isolates and y = 0 for oral isolates when looking for pathogenic patterns. For species prediction we used the labels: y = 1 for *S*. *sanguinis* and y = 0 for *S*. *gordonii*. Each model was trained and tested 100 times, where the prediction probability for the test set was averaged across the 100 predictions. Then the average probability across the 100 predictions was used to calculate the AUC and MCC. The ROC curve was illustrated based on a single run. The OneHotEncoder library from Scikit-learn v. 9.0.1^67^ was used for one-hot-encoding. Mean AUC and MCC were calculated using random labelling of the samples; the labels were shuffled for each of the 100 runs. A paired t-test was applied to the different models, to determine if the Random Forest performs significantly better on labelling based on pathogenesis and species than on random labelling.

### Cross validation

Cross validation (CV) was done using stratified 5-fold CV as well as a LOU CV. Based on 100 runs, we found the model to have a significant better performance when using the count of individual functional domains in each genome rather than the absence or presence (AP) of the protein families (100 runs: mean AUC count = 0.57, mean AUC AP = 0.55, t-test: p-value = 0.007). The LOU CV was found to perform significantly better than 5-fold CV (100 runs: mean AUC LOU = 0.58, mean AUC 5-fold = 0.56, t-test: p-value = 0.007). We therefore based the analyses on the LOU CV model using non-redundant datasets.

### Amino acid variation from the core-genome

All amino acid variations were extracted from the core-genome using snp-site v.2.4.0^70^, on the concatenated alignment file generated in the section ‘*Phylogeny and strain clustering’*. This was used as input to a Random Forest model, with the same settings as described in the section above. To account for amino acid substitutions the BLOSUM62 matrix was used for embedding^71^.

### Prediction of virulence genes

Basic Local Alignment Search Tool (BLAST) v2.2.31+^19^ was used to align all genes from the 59 genomes against known virulence genes. The Virulence Factors of Pathogenic Bacteria (VFDB) protein sequences of core dataset as well as the VFDB protein sequences of the full dataset were used as databases^20–22^ (downloaded March 5^th^ 2018). Only the best hit for each gene was considered, and the threshold for hits was a bit-score >200 and a sequence identity percent >50%. The VFDB hits were then categorized using the virulence factors provided by VFDB in the intra-genera VF’s comparison tables. Genes that could not be categorized using these tables were assigned a Cluster of Orthologous Groups of proteins (COGs)^23^ using egg-NOG-mapper^72,73^.

An additional database was constructed, containing 10 *S*. *pneumoniae* TIGR4 *cps4* genes. The genes were downloaded from NCBI as protein sequences, and the protein ID, Locus tag and gene name can be found in Table S3. The 59 *Streptococcus* genomes were blasted against the database in the same manner as described above. The BLAST results can be found in Table S3.

### Ethical statement

Recognition of the streptococcal strains is part of the routine diagnostic protocol at the Departments of Clinical Microbiology in the Capital region of Denmark, region Zealand and the region of Southern Denmark. The strains were analysed anonymously in a retrospective manner, and ethical approval and informed consent were thus not required.

According to the rules from the Danish National Committee on Health Research Ethics, projects performed as described are not to be approved by the committee. This assessment was made at the department and institutional level. Ethical approval and informed consent were thus not required. The study was approved by the Danish Data Protection Agency (Journal number 2012-41-0240).

## Supplementary information

Supplementary Figure S1.

Supplementary Table S1.

Supplementary Table S2.

Supplementary Table S3.

## Data Availability

The genomic raw read data is available from ENA with the accession PRJEB30467.
