# Supplementary material for: Similar genomic patterns of clinical infective endocarditis and oral isolates of Streptococcus sanguinis and Streptococcus gordonii
Source: Sci Rep. 2020 Feb 17;10:2728. doi: 10.1038/s41598-020-59549-4 (PMC7026040; doi:10.1038/s41598-020-59549-4)
Supplement: Supplementary file 1 — Supplementary Figure S1. [file 41598_2020_59549_MOESM1_ESM.pdf]

Supplementary Information

Similar genomic patterns of clinical infective endocarditis and oral isolates of *Streptococcus sanguinis* and *Streptococcus gordonii*

Katrine Højholt Iversen[1,2*], Louise Hesselbjerg Rasmussen[2*], Kosai Al-Nakeeb[1], Jose Juan Almagro Armenteros[1], Christian Salgård Jensen[2], Rimtas Dargis[2], Oksana Lukjancenko[3], Ulrik Stenz Justesen[5], Claus Moser[4], Flemming S. Rosenvinge[5], Xiaohui Chen Nielsen[2], Jens Jørgen Christensen[2,6†], Simon Rasmussen[7†]

[1]Department of Health Technology, Section for Bioinformatics, Technical University of Denmark, Kemitorvet, Building 204, 2800 Kgs. Lyngby, Denmark.
[2]The Regional Department of Clinical Microbiology, Slagelse Hospital, Ingemannsvej 46, 4200 Slagelse, Denmark.
[3]Clinical Microbiomics, Ole Maaløes vej 3, 2200, Copenhagen N.
[4]Department of Clinical Medicine, Rigshospitalet, Henrik Harpestrengsvej 4A, 2100 Copenhagen Ø, Denmark.
[5]Department of Clinical Microbiology, Odense University Hospital, J.B. Winsløws Vej 21, 2, 5000 Odense C, Denmark.
[6]Institute of Clinical Medicine, University of Copenhagen, Blegdamsvej 3B, 2200 Copenhagen N
[7]Novo Nordisk Foundation Center for Protein Research, Faculty of Health and Medical Sciences, University of Copenhagen, Blegdamsvej 3B, 2200 Copenhagen N, Denmark.

Correspondance: jejc@regionsjaelland.dk and simon.rasmussen@cpr.ku.dk

Supplementary Figure S1

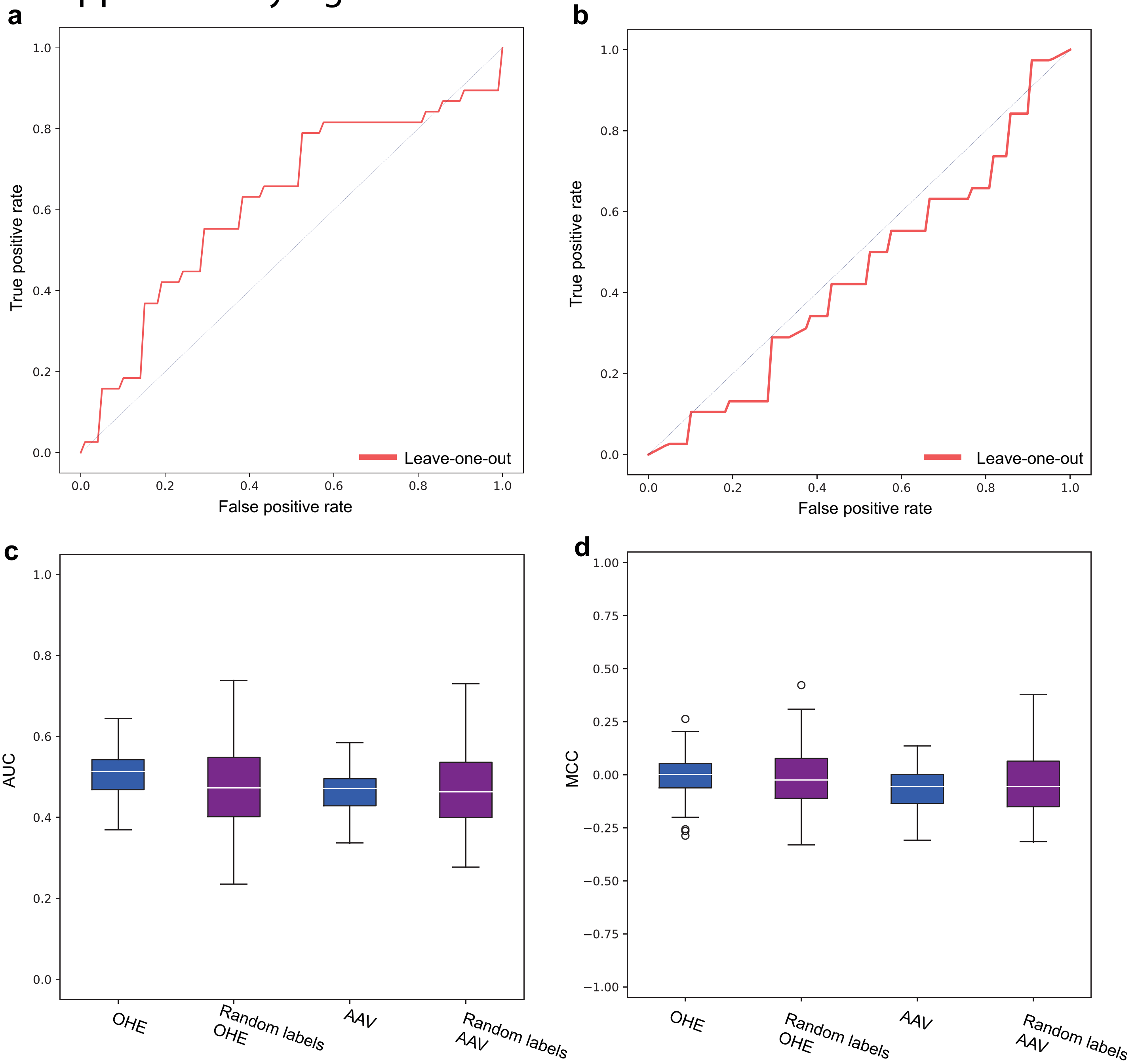

Supplementary Figure S1. Machine learning using Random Forest modelling with a leave-one-out cross-validation (LOU CV) to test if the model could distinguish between the IE and oral isolates. **A)** ROC curves using one-hot-encoding (OHE) based on the non-redundant count of individual protein domains in each genome (red). **B)** ROC curves based on the unique 21,165 amino acid variations (AAV) in the core-genome (red). **C)** Boxplots of the Area Under the Curve (AUC) determined from 100 runs using LOU CV. The blue boxplots illustrate AUC distributions of the OHE RF with on the non-redundant count of individual protein domains dataset. The purple boxplots illustrate the AUC distributions of the RF based on the 21,165 unique amino acid variations in the core-genome. **D)** Boxplots of Matthews Correlation Coefficient (MCC) determined from 100 runs using LOU CV. The blue boxplots illustrate MCC distributions of the OHE RF with the non-redundant count of individual protein domains dataset. The purple boxplots illustrate the MCC distributions of the RF based on the 21,165 unique amino acid variations in the core-genome. Both boxplots shows the distribution of the data by illustrating the minimum and maximum values, as well as the first and third quartile (the box) with the median highlighted (white). Outliers are illustrated as circles outside of the plot. The boxplots also show the values when random labelling is applied to the RF models.
